# Genomic patterns of homozygosity and inbreeding depression in Murciano-Granadina goats

**DOI:** 10.1186/s40104-022-00684-5

**Published:** 2022-03-10

**Authors:** María Gracia Luigi-Sierra, Almudena Fernández, Amparo Martínez, Dailu Guan, Juan Vicente Delgado, Javier Fernández Álvarez, Vincenzo Landi, Francesc Xavier Such, Jordi Jordana, María Saura, Marcel Amills

**Affiliations:** 1 Centre for Research in Agricultural Genomics (CRAG), CSIC-IRTA-UAB-UB, Universitat Autònoma de Barcelona, 08193 Bellaterra, Spain; 2 Departamento de Mejora Genética Animal, INIA, Carretera de la Coruña km 7,5, 28040 Madrid, Spain; 3 Departamento de Genética, Universidad de Córdoba, 14071 Córdoba, Spain; 4 Department of Veterinary Medicine, University of Bari ‘‘Aldo Moro”, 62 per Casamassima km. 3, 70010 Valenzano, SP Italy; 5 Group of Research in Ruminants (G2R), Department of Animal and Food Science, Universitat Autònoma de Barcelona (UAB), Bellaterra, Barcelona, Spain; 6 Departament de Ciència Animal i dels Aliments, Facultat de Veterinària, Universitat Autònoma de Barcelona, 08193 Bellaterra, Spain

**Keywords:** Goat, Inbreeding, Milk yield, Murciano-Granadina, Somatic cell score

## Abstract

**Background:**

Inbreeding depression can adversely affect traits related to fitness, reproduction and productive performance. Although current research suggests that inbreeding levels are generally low in most goat breeds, the impact of inbreeding depression on phenotypes of economic interest has only been investigated in a few studies based on genealogical data.

**Results:**

We genotyped 1040 goats with the Goat SNP50 BeadChip. This information was used to estimate different molecular inbreeding coefficients and characterise runs of homozygosity and homozygosity patterns. We detected 38 genomic regions with increased homozygosity as well as 8 ROH hotspots mapping to chromosomes 1, 2, 4, 6, 14, 16 and 17. Eight hundred seventeen goats with available records for dairy traits were analysed to evaluate the potential consequences of inbreeding depression on milk phenotypes. Four regions on chromosomes 8 and 25 were significantly associated with inbreeding depression for the natural logarithm of the somatic cell count. Notably, these regions contain several genes related with immunity, such as *SYK*, *IL27*, *CCL19* and *CCL21*. Moreover, one region on chromosome 2 was significantly associated with inbreeding depression for milk yield.

**Conclusions:**

Although genomic inbreeding levels are low in Murciano-Granadina goats, significant evidence of inbreeding depression for the logarithm of the somatic cell count, a phenotype closely associated with udder health and milk yield, have been detected in this population. Minimising inbreeding would be expected to augment economic gain by increasing milk yield and reducing the incidence of mastitis, which is one of the main causes of dairy goat culling.

**Supplementary Information:**

The online version contains supplementary material available at 10.1186/s40104-022-00684-5.

## Background

Inbreeding is defined as the mating of individuals that are related to each other more closely than the average relationship within the concerned population [1]. In livestock species, the magnitude of inbreeding has been traditionally measured through genealogical information [2]. However, pedigree-based estimates are affected by the depth of the pedigree [2] because founders are assumed to be unrelated and non-inbred [3]. Consequently, inbreeding produced by distant ancestors not included in the pedigree is systematically ignored [4]. Another disadvantage of quantifying inbreeding from pedigree data is that it provides bare expectations about the fraction of the genome which is identical-by-descent (IBD) [3]. With the advent of high-density arrays of single nucleotide polymorphisms (SNPs), it has become possible to estimate genomic inbreeding coefficients which circumvent these limitations [5]. Indeed, important advantages of genomic inbreeding coefficients over their genealogical counterparts are: (i) higher accuracy to differentiate among individuals within the same pedigree, since variation due to Mendelian sampling is captured [4], (ii) higher accuracy to quantify shared ancestry of genetic haplotypes [4], and (iii) the ability to map inbreeding to specific genomic regions [6]. Different types of genomic inbreeding coefficients have been implemented. While inbreeding coefficients based on the proportion of homozygous SNPs (*F*_*HOM*_) just reflect identity-by-state (IBS) allele-sharing proportions [6], coefficients (*F*_*ROH*_) based on measuring the fraction of the genome covered by runs of homozygosity (ROH) estimate IBD allele sharing [4–8], making possible to disentangle recent from ancient inbreeding [3–9].

The increase of inbreeding might have adverse consequences on the fitness of livestock populations due to the loss of genetic variability, which can entail a long-term reduction of genetic variance (due to the fixation of alleles) and, consequently, a slowing down of the rate of response to selection in breeding schemes [10–12]. Moreover, incremented levels of inbreeding might reduce the mean phenotypic performance of livestock populations, a phenomenon known as inbreeding depression (reviewed by Leroy [2]). Although inbreeding depression is particularly intense for fitness and reproduction traits [11], there is broad evidence that it also decreases dairy and growth performances [2, 13–15]. Besides, susceptibility to certain diseases, such as mastitis, is increased in inbred animals [16, 17]. In Holstein cattle, a 1% increase of inbreeding is expected to cause a reduction of $22–24 of lifetime net income per individual [18], while in sheep the average economic loss per ewe amounts to $17 for moderate inbreeding and $36 when inbreeding is close to 50% [18].

Several studies have used genomic methods to determine the levels of inbreeding in goat populations with a broad geographic distribution [19–21]. A recent investigation carried out by Bertolini et al. [19] revealed that short ROH (< 3 Mb) are particularly abundant in worldwide goat populations. Moreover, five regions on caprine chromosomes (CHI) 11, 12, and 18 contained ROH hotspots that overlapped with signatures of selection [19]. The majority of goat breeds analysed by Bertolini et al. [19] displayed low levels of inbreeding (*F*_*ROH*_ < 0.10), with the only exception of certain local breeds with small population sizes (e.g. Dutch Landrace goats) as well as of breeds with insular origins (e.g. Icelandic and Malagasy goats) which happened to be highly inbred [19, 20]. Despite the fact that inbreeding depression can have adverse effects on the profitability of farmers and animal breeders, very few studies have investigated its consequences on goat production [22, 23]. In this regard, Marete et al. [22] and Deroide et al. [23] estimated, with genealogical methods, the effect of inbreeding depression on the production of Kenya Alpine and Murciano-Granadina goats, respectively, and they found that in both populations such effect was negligible.

The goals of the current work were: (i) to measure the levels of inbreeding in a Murciano-Granadina resource population by using different genomic coefficients, and (ii) to use this information to infer the impact of inbreeding depression on dairy phenotypes recorded in this population.

## Methods

### Animal material and phenotyping

The animal material comprised 1040 Murciano-Granadina female goats from 15 farms located in the autonomous region of Andalusia (Spain). Murciano-Ganadina is a local Spanish breed officially created in 1975 by the crossbreeding of Murciano and Granadina goats [24]. Currently, it has a census of 115,105 heads (2020), and its remarkable adaptability to harsh environments as well as its good milking performance (mean of 586 kg/lactation; 5.1% of fat and 3.6% of protein in milk) have made it a very popular breed in Spain and other countries (https://www.mapa.gob.es/es/ganaderia/ temas/zootecnia/razas-ganaderas/razas/catalogo-razas/caprino/murciano-granadina/).

Blood samples were extracted from goats using vacuum tubes coated with EDTA K_3_ anticoagulant and stored at − 20 °C until processing. Phenotypic records for milk yield and composition traits were recorded in the framework of the selection program of the Murciano-Granadina goat breed [24]. Only phenotypes corresponding to the first parity (recorded between the years 2009 and 2017) were taken into consideration. The following phenotypes were recorded in 817 goats: milk yield measured in kilograms at 210 days (MY210), 240 days (MY240) and 305 days (MY305), the natural logarithm of the somatic cell count divided by 1000 (lnSCC, to convert this value into a somatic cell count please use the formula: e^lnSCC^ × 10^3^ cells/mL), fat percentage (FP), protein percentage (PP) and lactose percentage (LP). Milk composition traits were standardised to a lactation of 210 days. Summary statistics of phenotypic records are displayed in Table 1.

**Table 1:** Summary statistics of seven milk production and composition traits recorded in 817 Murciano-Granadina goats

Traits^a^	Mean	SD
MY210, kg	395.647	131.787
MY240, kg	450.493	142.707
MY305, kg	547.418	179.840
lnSCC	6.278	0.937
FP, %	5.190	0.766
PP, %	3.563	0.351
LP, %	4.865	0.228

^**a**^*MY210*, milk yield at 210 days of lactation (kg); *MY240*, milk yield at 240 days of lactation (kg); *MY305*, milk yield at 305 days of lactation (kg); *lnSCC*, natural logarithm of the somatic cell count divided by 1000 (to convert this value into a somatic cell count use the formula: e^lnSCC^ × 10^3^ cells/mL); *FP*, fat percentage; *PP*, protein percentage; *LP*, lactose percentage

### Generation of high throughput genotypic data

The isolation of genomic DNA was carried out following a salting-out protocol [25]. Three mL of whole blood were mixed with 4 volumes of Red Cell Lysis Solution (Tris-HCl 10 mmol/L, pH = 6.5; EDTA 2 mmol/L; Tween 20 1%), and this mixture was centrifuged at 2000 × *g*. The supernatant was discarded and the pellet was resuspended in 3 mL of lysis buffer (Tris-HCl 200 mmol/L, pH = 8, EDTA 30 mmol/L, SDS 1%; NaCl 250 mmol/L) plus 100 μL proteinase K (20 mg/mL) and incubated for 3 h at 55 °C. The lysate was chilled, and 1 mL of ammonium acetate 10 mol/L was added to it. After centrifugation at 2000 × *g* for 10 min, the supernatant (~ 4 mL) was transferred to a new tube with 3 mL of isopropanol 96%, and this mixture was centrifuged at 2000 × *g* for 3 min. The resulting DNA pellet was washed with 3 mL of ethanol 70% and an additional centrifugation step at 2000 × *g* for 1 min was performed. The DNA pellet was dried at room temperature, and it was subsequently resuspended in 1 mL of TE buffer (Tris-HCl 10 mmol/L, EDTA 1 mmol/L, pH = 8). Murciano-Granadina goats were genotyped with the Goat SNP50 BeadChip (Illumina Inc., San Diego, CA, USA) by following the instructions of the manufacturer. The goat ARS1 genome [26] was used as reference for inferring the genomic location of the SNPs, and the position and the name of each SNP were updated using the PLINK 1.9 software [27]. Only individuals with at least 95% of SNPs with genotype calls were taken into consideration. Moreover, only SNPs meeting the following requirements were used in the downstream analyses: (i) mapping to autosomes, (ii) displaying a minor allele frequency of 0.05 or higher, (iii) not deviating significantly (*P* < 0.00001) from the Hardy-Weinberg equilibrium, and (iv) with a genotype call rate over 98%. Data were filtered using PLINK 1.9 [27]. In addition, a principal component analysis (PCA) was carried out with PLINK 1.9 [27] in order to assess population structure. The visualisation of such results was based on the first two components of the PCA.

### Detection of runs of homozygosity

The definition of ROH followed six criteria: (i) the minimum length of ROH is 1 Mb, (ii) a ROH must contain at least 15 SNPs, (iii) the density of SNPs per ROH was set to at least 1 SNP every 100 kb, (iv) the maximum distance between consecutive SNPs is 250 Kb, (v) one heterozygous position per ROH is allowed, and (vi) one missing position per ROH is allowed. Said criteria were established based on the density of the genotyping panel, with a mean distance between consecutive SNPs of 51.73 Kb and a mean number of 19.35 SNPs/Mb (Additional file 1: Table S1), and several of them are based on the ROH definition established by the AdaptMap Consortium [19, 20]. Runs of homozygosity were identified with the PLINK 1.9 software [27] using a sliding window of 50 SNPs.

### Analysis of the genomic patterns of homozygosity

The proportion of homozygosity per site was estimated as the ratio between the number of animals with homozygous genotypes for a particular SNP divided by the number of animals genotyped for that SNP. A sliding window encompassing 30 SNPs was designed to estimate the average of this ratio, and chromosomal patterns of homozygosity were visualised as Manhattan plots using R [28].

Genomic coverage and distribution of ROH were also investigated. The patterns of ROH size and distribution along the genome were analysed and plotted using R [28]. Genomic regions in which ROH are prevalent, the so-called ROH hotspots, were identified by measuring the proportion of animals that harbour a particular SNP occurring within a ROH with regard to the total number of animals genotyped for that SNP. Genomic regions containing the top 1% SNPs most commonly associated with ROH were classified as ROH hotspots [29, 30]. Both highly homozygous regions and ROH hotspots were compared, and overlapping segments were identified. Taking as reference the ARS1 goat genome [26], genes mapping to these overlapping segments were extracted with the Biomart tool of Ensembl [31]. Database for Annotation, Visualization and Integration Discovery (DAVID) Bioinformatics Resources [32] and UniProt [33] were used in Gene ontology analysis to identify over-represented (enriched) gene ontology (GO) terms and KEGG pathways. Amongst other things, DAVID provides biological context for long lists of genes by assigning them to functionally related groups through the use of fuzzy clustering techniques [32]. To assess the significance of gene-enrichment in annotation terms, a Fisher Exact is employed. Only terms with Max. Prob ≤ 0.1, Min. Count ≥ 2 and *P* < 0.05 were considered as significantly enriched [32].

### Estimation of inbreeding coefficients

Six inbreeding coefficients were estimated at the whole genome level based on genotypic data:

*F*_*HOM*_ is defined as the proportion of SNPs with homozygous genotypes [6] and was estimated as:

$$ {F}_{HOM_i}=\frac{H_{O_i}}{S} $$

where *H*_*Oi*_ corresponds to the observed number of homozygous genotypes for all the SNPs for each individual *i* and *S* are the number of SNPs for which individual *i* has genotype data. It was computed from the output of the --het command of PLINK 1.9 software [27].

*F*_*ROH*_ was estimated as the proportion of the genome covered by ROH by using the following formula:

$$ {F}_{ROH_i}=\frac{\sum {L}_{ROH_i}}{L_{auto}} $$

where *L*_*ROHi*_ corresponds to the sum of the lengths of all ROH present in each individual *i*, and *L*_*auto*_ is the total length of the autosomal goat genome covered by SNPs [5]. The same mathematical expression was used to calculate the genomic coverage of ROH with sizes smaller (*F*_*ROHShort*_) or larger (*F*_*ROHLong*_) than 5 Mb. Such calculations were made to assess the relative importance of distant (*F*_*ROHShort*_) versus recent (*F*_*ROHLong*_) inbreeding [6], according to the size of the genome and SNP density (Table 1). Assuming that the length of the ROH segments follows an exponential distribution with a mean equal to $\frac{1}{2}g$, where *g* corresponds to the number of generations to the closest common ancestor [34], and also assuming that goats have a recombination rate of approximately 1 cM/Mb [35], *F*_*ROHShort*_ indicates the inbreeding of an individual from 10 to 50 generations in the past, while *F*_*ROHLong*_ estimates the inbreeding from 1 to 9 generations in the past.

*F*_*IS*_ coefficient of Wright [36] was calculated with the formula:

$$ {F}_{IS}=\frac{H_{E_i}-{H}_{O_i}}{H_{E_i}} $$

Where ${H}_{E_i}$ and ${H}_{O_i}$ are the expected and observed heterozygosities of the individual *i*. This coefficient was estimated from the output of the --hardy command of PLINK 1.9 [27].

*F*_*YANG*_ coefficient was estimated from the diagonal of the matrix of genomic relationships of Yang based on the correlation between uniting gametes [37, 38]. It was calculated with the following formula:

$$ {F}_{YANG_i}=\frac{1}{S}\sum \limits_{k=1}^s\frac{x_k^2-\left(1+2{p}_k\right){x}_{k_i}+2{p}_k^2}{2{p}_k\left(1-{p}_k\right)} $$

where, *x*_*k*_ is the genotype of the individual *i* for the SNP *k*, and *p*_*k*_ is the frequency of the reference allele in the studied population. The command --ibc of PLINK 1.9 [27] was used to estimate it.

The coefficients *F*_*HOM*_ and *F*_*ROH*_ were also estimated at the chromosomal level.

### Inbreeding depression analyses

The effects of inbreeding depression on dairy traits were investigated using data from 817 goats with available phenotypes (MY210, MY240, MY305, lnSCC, FP, PP and LP). Analyses were performed with the REMLF90 software [39] to implement a restricted maximum likelihood (REML) analysis approach, in which the phenotypic values of each trait in each individual are regressed onto its inbreeding coefficient using a linear mixed model. These analyses were performed to quantify inbreeding depression at the whole genome scale as well as on a chromosome and regional basis. The model was fitted as follows:

$$ y= X\beta + Za+e $$

where *y* is the vector of observations for each phenotype, *β* is a vector of fixed effects, including farm (15 levels), year of birth (10 levels), and the linear regression on *F* as a covariate; *a* is the vector of additive genetic effects, *e* is the vector of random residual effects, and *X* and *Z* are incidence matrices relating fixed and random effects to observations.

The significance of the inbreeding effect on the analysed traits was determined by applying a two-tailed hypothesis test. A Z-statistic was estimated with the following general formula:

$$ Z=\frac{\overline{\mathbf{x}}-{\mu}_0}{s.e.} $$

Here, $\overline{x}$ corresponds to the regression coefficient representing the effect of inbreeding over each trait, and *μ*_*0*_ is the coefficient of inbreeding corresponding to the null hypothesis (in this case is equal to 0), and *s.e*. is the standard error. The transformation of Z-scores into *P*-values was accomplished with the function *pnorm()* implemented on R [28].

The above analysis was performed by regressing each phenotype onto four genomic inbreeding coefficients (*F*_*HOM*_, *F*_*ROH*_, *F*_*ROHLong*_, and *F*_*ROHShort*_). In order to detect genomic regions associated with inbreeding depression, analyses at the chromosomal level were performed for traits that in the whole-genome analysis were identified as significantly affected by inbreeding depression (*P* < 0.05). Following Saura et al. [6], inbreeding depression was finely mapped by dividing chromosomes into six segments and performing the analyses reported above in each segment. As previously explained, genes mapping to genomic regions associated with inbreeding depression for a specific trait were retrieved using Biomart [31], and their biological functions were assessed with UniProt [33] and David Bioinformatics Resources version 6.8 [32].

## Results

### Assessment of homozygosity patterns in Murciano-Granadina goats

**Fig. 1:**
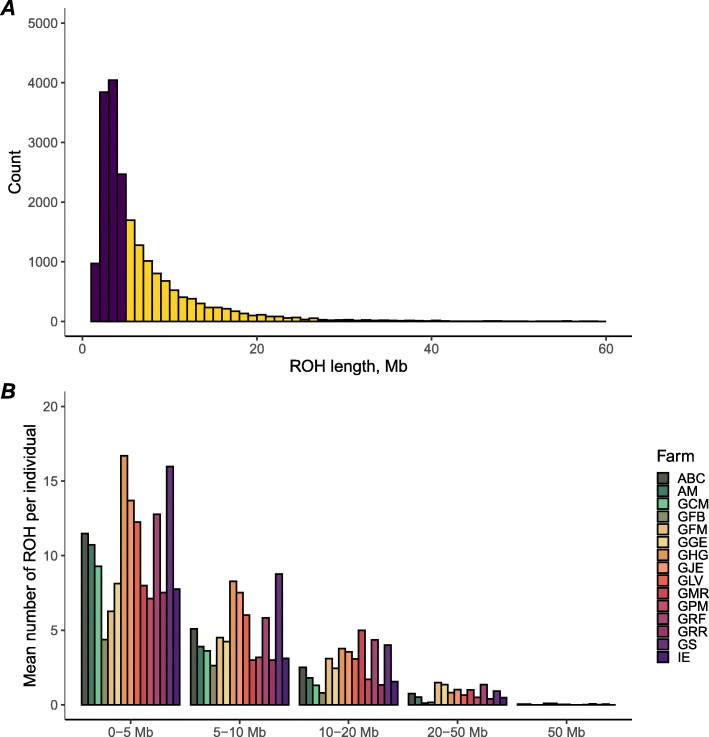
**A** Number of ROH classified according to their length. Purple and yellow bars represent the counts of ROH shorter and longer than 5 Mb, respectively. **B** Number of ROH classified according to their length category by the farm of origin

**Fig. 2:**
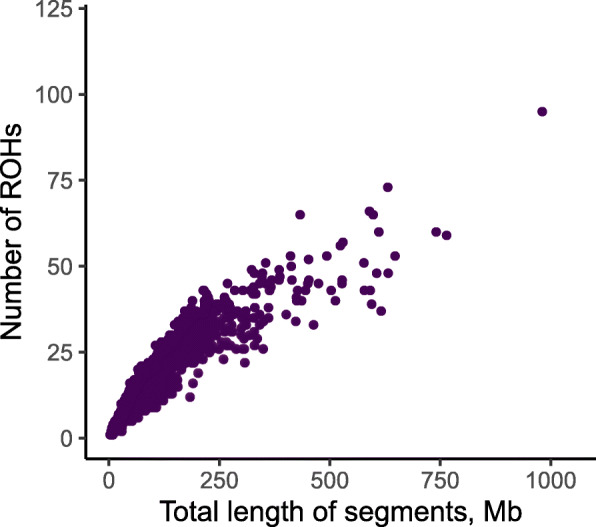
Relationship between ROH number and length in 1040 Murciano-Granadina goats genotyped with the Goat SNP50 BeadChip

A total of 46,689 SNPs and 1040 animals were selected to investigate the population structure and patterns of homozygosity of Murciano-Granadina goats. The PCA (Additional file 2: Fig. S1) indicated that goats clustered, to some extent, in accordance with their farm of origin. We detected 20,312 ROH that were classified as follows: 11,325 had sizes of 0–5 Mb, while 5470 (5–10 Mb), 2695 (10–20 Mb), 789 (20–50 Mb) and 33 (> 50 Mb) displayed sizes above 5 Mb. The mean number of ROH per category and per individual varied slightly across farms (Fig. 1A and B). The mean ROH number was 19.53 ± 11.89 per individual, with an average length of 6.15 ± 2.05 Mb. As depicted in Fig. 2, the majority of the individuals harboured less than 50 ROH, and ROH covered a small proportion of the genome (< 300 Mb, about 10% of the genome). Only 2% of the individuals showed a genomic ROH coverage > 500 Mb, and 1% harboured more than 50 ROH. Larger chromosomes encompassed a greater number of ROH when compared to the smaller ones (Additional file 3: Fig. S2), and the correlation coefficient between the number of ROH and chromosome length was 0.92 (*P* < 0.05).

**Fig. 3:**
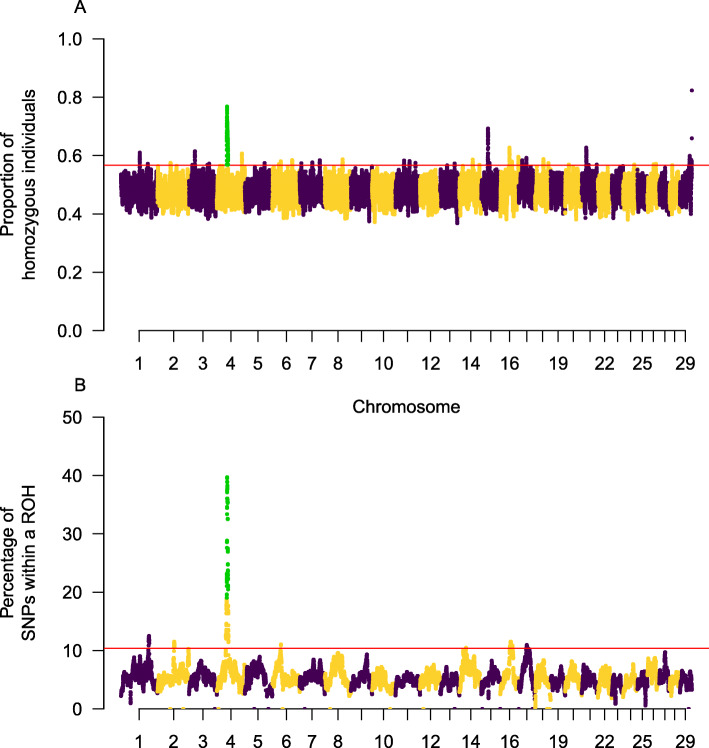
**A** Proportion of individuals with homozygous genotypes for each SNP marker. The *y*-axis displays the proportion of individuals for which a specific SNP displays a homozygous genotype, while the *x*-axis corresponds to the positional coordinates of SNPs distributed in the 29 caprine autosomes. **B** ROH hotspots identified in the population of Murciano-Granadina goats under study. The *y*-axis displays the frequency at which a given SNP is found within a ROH in the population; while the *x*-axis corresponds to the positional coordinates of SNPs distributed in the 29 caprine autosomes. Markers above the red line are in the top 1% of each category (homozygosity or frequency of being within a ROH). Markers highlighted in green are located in genomic regions consistently identified as regions of high homozygosity and ROH hotspots

The genome-wide analysis of homozygosity, based on the proportion of homozygous individuals for each genotyped position, made it possible to detect 38 genomic regions with increased homozygosity (harbouring the top 1% of the most homozygous positions) that were scattered on 20 goat chromosomes (CHI), i.e. CHI 1–8, 11, 13–18, 20, 21, 24, 26 and 29 (Fig. 3A and Additional file 4: Table S2). Eight ROH hotspots mapping to CHI 1, 2, 4, 6, 14, 16, and 17 were identified (Fig. 3B and Additional file 5: Table S3). One region (i.e. CHI4:42,552,375–48,378,207 bp) was consistently detected in the genome-wide analysis of homozygosity and ROH. Sixty-six genes mapped to these regions (Additional file 6: Table S4) and a functional enrichment analysis revealed 15 GO terms significantly enriched at the nominal level (*P* < 0.05). Particularly significant were GO terms related with ferric and copper import into the cell (Additional file 7: Table S5).

### Estimation of inbreeding coefficients

**Fig. 4:**
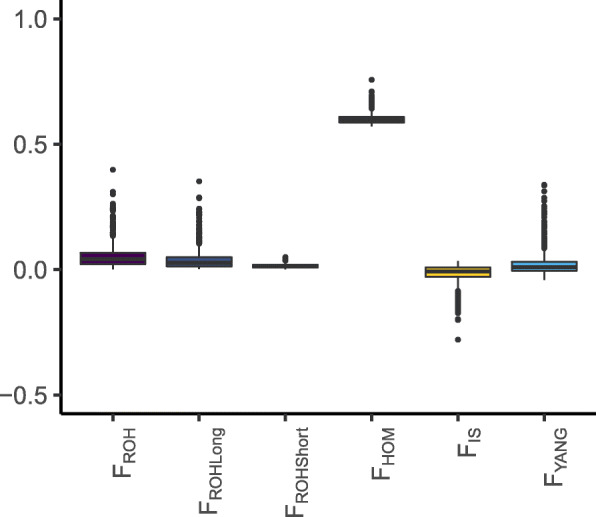
Boxplots depicting the magnitude and dispersion of molecular inbreeding *F*_*ROH*_, *F*_*ROHLong*_, *F*_*ROHShort*_, *F*_*HOM*_*, F*_*IS*_ and *F*_*YANG*_ coefficients estimated in 1040 female Murciano-Granadina goats. Differences in magnitude between *F*_*HOM*_ and the other molecular coefficients are due to the fact that they indicate identity-by-state and identity-by-descent allele-sharing proportions, respectively

Genomic inbreeding coefficients reached values of 0.601 ± 0.021 (*F*_*HOM*_); 0.053 ± 0.046 (*F*_*ROH*_); 0.040 ± 0.041 (*F*_*ROHLong*_); 0.014 ± 0.008 (*F*_*ROHShort*_); − 0.016 ± 0.035 (*F*_*IS*_) and 0.023 ± 0.047 (*F*_*YANG*_). The magnitude and dispersion of these coefficients are shown in Fig. 4. The *F*_*HOM*_, *F*_*ROH*_, *F*_*ROHLong*_, *F*_*IS*_ and *F*_*YANG*_ coefficients were highly correlated, being especially high the correlations between *F*_*HOM*_ and *F*_*ROH*_, *F*_*ROH*_ and *F*_*ROHLong*_ (*r* = 0.99, *P*< 2.2 × 10^−16^) and between *F*_*HOM*_ and *F*_*IS*_ (*r* = − 1, *P* < 2.2 × 10^−16^). In contrast, *F*_*ROHShort*_ showed the weakest correlations with the remaining inbreeding coefficients (|*r| =* 0.33–0.64), although their statistical significance (*P* < 2.2 × 10^−16^) was very high (Table 2).

**Table 2:** Pearson correlations between molecular inbreeding coefficients (*F*) estimated in 1040 Murciano-Granadina goats

***F*** coefficient	***F***_***HOM***_	***F***_***ROH***_	***F***_***ROHLong***_	***F***_***ROHShort***_	***F***_***IS***_	***F***_***YANG***_
***F***_***HOM***_	1					
***F***_***ROH***_	0.99	1				
***F***_***ROHLong***_	0.97	0.99	1			
***F***_***ROHShort***_	0.64	0.59	0.45	1		
***F***_***IS***_	– 1	– 0.99	– 0.97	– 0.64	1	
***F***_***YANG***_	0.86	0.89	0.92	0.33	– 0.86	1

*All correlation coefficients (lower part of the matrix) were significant (*P* < 2.2 × 10^−6^)

### Measurement of inbreeding depression for dairy traits

The natural logarithmic transformation of the somatic cell count divided by 1000 (lnSCC) as well as milk yield at three different time points (MY210, MY240 and MY305) showed significant evidence of inbreeding depression when analysing *F*_*HOM*_ and *F*_*ROH*_. In contrast, no significant effects of inbreeding were identified for coefficients based either on short or long ROH. Increases of 0.1 units of *F*_*HOM*_ and *F*_*ROH*_ coefficients involved lnSCC increments of 0.29 (*P* *=* 0.037) and 0.127 (*P* *=* 0.038) units, respectively (Table 3). At the chromosomal level, significant inbreeding depression for lnSCC was detected on CHI8 and CHI25 when regressed on either *F*_*HOM*_ or *F*_*ROH*_*,* while six additional chromosomes (CHI13, CHI14, CHI22, CHI24, CHI25 and CHI27) displayed inbreeding depression for this trait exclusively when it was regressed onto *F*_*HOM*_ (Additional file 8: Table S6). Four regions containing 666 genes on chromosomes 8 (i.e. CHI8:37,557,623–56,336,435 bp and CHI8:75,115,244–93,894,055 bp) and 25 (i.e. CHI25: 82,419–7,143,084 bp and CHI25:21,429,255–28,572,340 bp) displayed a significant inbreeding depression for lnSCC based on *F*_*HOM*_ (Additional file 9: Table S7 and Additional file 10: Table S8). After performing functional enrichment analysis, various GO terms and pathways involved in the immune response were significantly enriched only at the nominal level, including integrin domains (i.e. *ITGAM*, *ITGAD*, *ITGAX*, *ITGAL*) and genes with protein kinase activity chemotaxis and cell signalling activities (i.e. *CCL19*, *CCL21*, *CCL24*, *CCL26*, *SYK* and *IL27*). Several of these genes display functions related with innate immunity and inflammatory response e.g. lymphocyte chemotaxis, cellular response to interferon-γ, immunological synapse formation, positive regulation of chemotaxis, cellular response to interleukin-1, monocyte chemotaxis etc. (Additional file 11: Table S9).

**Table 3:** Inbreeding depression estimates for milk yield and composition traits expressed as the change of the phenotypic mean per 0.1 units increase of the corresponding inbreeding coefficient (95% confidence intervals are displayed in brackets)

Trait^**b**^	***F***_***HOM***_		***F***_***ROH***_		***F***_***ROHLong***_		***F***_***ROHShort***_	
	Mean ± s.e.^**a**^ (CI 95%)	***P***-value	Mean ± s.e.^**a**^ (CI 95%)	***P***-value	Mean ± s.e.^**a**^ (CI 95%)	***P***-value	Mean ± s.e.^**a**^ (CI 95%)	***P***-value
**MY210**	– 48.503 ± 21.124 (− 89.906 to – 7.101)	0.011	– 20.492 to ±9.356 (− 38.830 to – 2.154)	0.014	21.231 ± 25.901 (− 29.536 to 71.998)	0.206	19.751 ± 83.308 (− 143.533 to 183.034)	0.406
**MY240**	– 51.017 ± 24.159 (− 98.369 to – 3.666)	0.017	– 21.296 to ±10.702 (− 42.272 to – 0.320)	0.023	34.358 ± 30.841 (− 26.090 to 94.806)	0.133	10.158 ± 93.948 (− 173.980 to 194.296)	0.457
**MY305**	– 55.719 ± 29.987 (− 114.493 to 3.055)	0.032	– 23.196 to ±13.284 (− 49.233 to 2.841)	0.040	48.052 ± 47.583 (− 45.211 to 141.315)	0.156	– 16.834 ± 118.042 (− 248.196 to 214.527)	0.443
**lnSCC**	0.290 ± 0.162 (− 0.027 to 0.608)	0.037	0.127 ± 0.072 (− 0.013 to 0.268)	0.038	– 0.268 ± 0.635 (− 1.514 to 0.977)	0.336	– 0.368 ± 0.649 (− 1.640 to 0.905)	0.286
**FP**	0.119 ± 0.121 (− 0.117 to 0.356)	0.161	0.046 ± 0.053 (− 0.059 to 0.150)	0.196	– 0.016 ± 0.209 (− 0.426 to 0.394)	0.469	– 0.031 ± 0.428 (− 0.871 to 0.809)	0.471
**LP**	0.029 ± 0.044 (− 0.057 to 0.116)	0.252	0.015 ± 0.019 (− 0.024 to 0.053)	0.226	– 0.090 ± 0.172 (− 0.428 to 0.247)	0.300	– 0.073 ± 0.169 (− 0.404 to 0.257)	0.332
**PP**	0.071 ± 0.068 (− 0.062 to 0.204)	0.147	0.035 ± 0.030 (− 0.024 to 0.093)	0.122	0.039 ± 0.106 (− 0.169 to 0.246)	0.358	– 0.157 ± 0.257 (− 0.660 to 0.346)	0.270

^a^s.e. standard error; CI 95, 95% confidence interval

^*b*^*MY210*; milk yield at 210 days of lactation (kg); *MY240*; milk yield at 240 days of lactation (kg); *MY305*; milk yield at 305 days of lactation (kg); *lnSCC*, natural logarithm of the milk somatic cell count divided by 1000 (to convert this value into a somatic cell count use the formula: e^lnSCC^ × 10^3^ cells/mL); *FP*, milk fat percentage (%); *LP*, milk lactose percentage (%); *PP*, milk protein percentage (%)

* numbers in bold correspond to *P*-values below 0.05

With regard to the milk yield traits, an increment of 0.1 units of *F*_*HOM*_ or *F*_*ROH*_ involved a decrease of 48.50 kg (*P* = 0.011) and 20.49 kg (*P* = 0.014) of milk for MY210; 51.02 kg (*P* = 0.017) and 21.30 kg (*P* = 0.023) for MY240; and 55.72 kg (*P* = 0.032) and 23.20 kg (*P* = 0.040) for MY305, respectively (Table 3). The analysis at the chromosomal level indicated significant inbreeding depression for MY210 and MY240 on CHI2, CHI3 and CHI11, while for MY305 inbreeding depression was significant only on CHI2 (Additional file 12: Table S10). The region on CHI2, comprised between 22,751,824 and 68,255,473 bp displayed significant inbreeding for all three milk yield traits (Additional file 13: Table S11). A total of 355 genes mapped to this genomic region (Additional file 14: Table S12). The functional enrichment analysis evidenced an overrepresentation of genes involved in mitochondrial and energetic processes, including genes from the PPAR signalling pathway i.e. *CYP27A1*, *ACADL*, *DBI* and *ACSL9* (Additional file 15: Table S13).

Although there is no substantial overlap between regions associated with inbreeding depression and ROH hotspots, the CHI8:75–93.8 Mb region associated with inbreeding depression for lnSCC and the CHI2:45.5–68.25 Mb region associated with inbreeding depression for milk yield show positional concordance with two of the 38 regions displaying high homozygosity (i.e. CHI8:76.25–77.4 Mb and CHI2:56.13–57.17 Mb).

## Discussion

### Low inbreeding in Murciano-Granadina goats

For the majority of Murciano-Granadina goats, ROH number and total length were below 50 and 350 Mb, respectively. Moreover, short ROH (< 5 Mb) were more abundant than the medium or long ones (Fig. 1A). These patterns are pretty consistent with what has been observed by Bertolini et al. [19] in a worldwide sample of goat breeds. Indeed, Bertolini et al. [19] showed that goat breeds from Southern Europe had, on average, 49 ROH per individual while the genomic coverage per individual was 183.47 Mb. In contrast, breeds from Northern Europe showed higher levels of homozygosity with 98 ROH per individual and genomic coverage of 479.17 Mb.

The inbreeding coefficients *F*_*ROH*_, *F*_*ROHShort*_, and *F*_*ROHLong*_ of Murciano-Granadina goats were mainly in the range of 0 to 0.05. In their study, Bertolini et al. [19] reported that about 60% of a worldwide sample of goat breeds displayed low *F*_*ROH*_ coefficients (< 0.10), while the remaining ~ 30% and ~ 10% of breeds showed moderate (0.10 < *F*_*ROH*_ < 0.20) or high (> 0.20) *F*_*ROH*_ values. The patterns of low homozygosity that we have observed in Murciano-Granadina goats contrast strongly with what has been reported in certain local breeds, such as Mallorquina, Pyrenean, and Valdostana, which have undergone sharp population bottlenecks [19, 20]. Low inbreeding and homozygosity in the Murciano-Granadina breed are probably explained by its large census size (> 100,000 individuals registered in the herdbook), the absence of population bottlenecks, and its broad geographic distribution encompassing more than 4000 farms across Spain (https://www.mapa.gob.es/es/ganaderia/temas/zootecnia/razas-ganaderas/razas) and other countries. Noteworthy, the Murciano-Granadina breed was founded by crossing, during the 1970s, two Murciana and Granadina populations with different historical origins [40]. Although genetic differentiation between these two populations was weak [40], this admixture event probably contributed to increase the heterozygosity of the resulting Murciano-Granadina breed. Widespread use of artificial insemination in reproductive management and intensive selection were implemented in the Murciano-Granadina breed a few decades ago [24], so their impact on genetic diversity and inbreeding has probably been quite limited so far.

While correlations between *F*_*HOM*_, *F*_*ROH*_, *F*_*ROHLong*_, *F*_*IS*_ and *F*_*YANG*_ were high, *F*_*ROHShort*_ displayed the lowest correlations with the remaining inbreeding coefficients, in line with previous studies focused on other livestock species [6, 13, 41]. Short ROH are mainly derived from ancient inbreeding events [5, 42] and do not reflect the whole autozygosity of the sample. It is also possible that several of these homozygous tracks are identical by state and not by descent, being produced by a low recombination rate or high linkage disequilibrium in unrelated ancestors [30]. Besides, when working with medium density genotype arrays (e.g. 50 K SNPs) the detection of short ROH can become quite difficult [43], thus limiting the ability to infer the true proportion of short vs. long ROH in the genome.

### Several ROH hotspots are detected in the genomes of Murciano-Granadina goats

We have identified several genomic regions in which ROH are particularly frequent (ROH hotspots). Similar patterns were found when the proportion of homozygous individuals was analysed at a genome-wide level i.e. 35.46% of the most common homozygous regions overlapped with ROH hotspots and these overlapping regions represented 35.42% of the total ROH hotspots. No positional coincidence was detected between ROH hotspots identified by us and those reported by Bertolini et al. [19] in a worldwide sample of goat populations. This finding agrees well with what has been previously observed in sheep [44]. Indeed, ROH hotspots are produced by factors such as positive selection and inversions suppressing recombination, that can differ substantially from population to population [42]. For instance, the characterisation of the products of 5860 female meioses in *Drosophila melanogaster* by genotyping more than 100 million SNPs made it possible to detect 106,964 recombination events displaying a remarkable degree of intra-specific variation [45]. Factors such as GC content, gene density, distribution of simple repeats and transposable elements, structural variation, and the presence of diverse poorly-characterised sequence motifs might explain the regional variation of the recombination rate across individuals and populations [46].

### ROH hotspots contain genes with diverse functions

Regarding the gene content of genomic segments co-localising with both high homozygosity regions and ROH hotspots (CHI4:42,552,375–48,378,207 bp), the functional enrichment analysis highlighted several gene ontology terms with nominally significant enrichment (*P*-value < 0.05) (Additional file 7: Table S5). From this list of genes, it is worth emphasising *STEAP1*, *STEAP2* and *STEAP4* that encode homonymous metalloreductases which facilitate the cellular uptake of iron and copper [47]. These proteins modulate the effects of intracellular oxidative stress and inflammation and are involved in multiple biological pathways related with molecular trafficking in the endocytic and exocytic pathways, metabolism, control of cell proliferation and apoptosis and tumour progression [48]. We also found genes with metabolic functions such as insulin-like growth factor binding protein 3 (*IGFBP3*) and insulin-like growth factor binding protein 1 (*IGFBP1*) that participate in the growth and postnatal development of cattle [49, 50]. These results are quite concordant with those reported by Mastrangelo and colleagues [30], who showed that ROH islands identified in Italian bovine breeds contained genes with heterogeneous functions related to milk production, reproduction, immune response, and resistance/susceptibility to infection and diseases.

### Effect of inbreeding depression on milk traits

The lnSCC in the Murciano-Granadina population under study averaged 6.25 ± 0.93 units, which is higher than values reported in primiparous goats from the Alpine (5.09 ± 1.36 units) and Saanen (5.32 ± 1.19 units) breeds [51]. Compared with cows and ewes, goats display higher numbers of somatic cells in milk. Indeed, the apocrine nature of milk secretion in goats increases the proportion of cytoplasmatic particles in milk, a feature, that depending on the measurement method of choice,  might increase the somatic cell count [52]. Besides, somatic cell count is modulated by many factors including the occurrence of bacterial infections, stress, oestrous cycle phase, diet etc. [53]. According to our results (Table 3), inbreeding depression increased lnSCC, a feature that is considered adverse because high lnSCC values are often associated with subclinical and clinical mastitis [51, 52].

The magnitude of inbreeding depression for lnSCC estimated from *F*_*ROHShort*_ strongly differed from estimates based on the other inbreeding coefficients (Table 3), a finding consistent with the moderate (|*r| =* 0.33–0.64) correlations between *F*_*ROHShort*_ and other molecular inbreeding coefficients (Table 2). Noteworthy, recent rather than ancestral inbreeding is the main cause of inbreeding depression in mammalian populations [54, 55]. Besides, long stretches of homozygosity usually contain a higher proportion of deleterious mutations than the shorter ones because they are more recent, so deleterious variation has not been yet purged by purifying selection [56].

In Murciano-Granadina goats, a previous study performed by Deroide et al. [23] reported a low percentage of inbreeding (average *F* = 0.24%). Milk production showed a positive quadratic correlation with inbreeding levels, but such effect was not significant. Deroide et al. [23] also reported that milk fat and dry extract contents experienced a slight increase due to inbreeding. In our study, the dairy trait mostly affected by inbreeding depression was lnSCC. Consistently, Doekes et al. [13] reported that a 1% increase of *F*_*ROH*_ involved a 0.86 ± 0.28 unit increase in somatic cell score (days 150 through to 400) recorded in Dutch Holstein-Friesian dairy cattle. In Iranian cattle, individuals with high inbreeding coefficients tended to have higher somatic cell scores than animals with low inbreeding coefficients [57], and similar results have been reported for Canadian Holstein cattle [58]. Doekes et al. [13] indicated that ancient inbreeding was the main contributor to inbreeding depression for somatic cell score, although such effect was not significant. Somatic cell score is an indicator of the health status of the mammary gland and substantial increases are observed in individuals suffering from mastitis [59]. Inbreeding has been reported to significantly reduce resistance against pathogens in multiple organisms [60–62], so the significant inbreeding depression observed for lnSCC in Murciano-Granadina goats might be explained, at least in part, by the weakening of the immune defences of the mammary gland. Thus, homozygosity for deleterious mutations might result in the partial or total inactivation of genes related with immunity, and low variability might also compromise the effectiveness of the immune response [63].

### Genomic regions associated with inbreeding depression for lnSCC contain several genes related with immunity

When we investigated which enriched clusters are present in the set of genes mapping to chromosomes 8 (37–56 Mb and 75–93 Mb) and 25 (0.082–7 Mb and 21–28 Mb) regions associated with inbreeding depression for lnSCC (Additional file 10: Table S8), we found several genes assigned to gene ontologies highly connected with immunity, e.g. integrin-mediated signalling pathway, lymphocyte chemotaxis, monocyte chemotaxis, immunological synapse formation, chemokine activity, etc. (Additional file 11: Table S9). The spleen tyrosine kinase (SYK) protein forms part of the integrins cluster and maps to CHI8: 86,755,291–86,861,895 bp (Additional file 11: Table S9). One of the functions of the SYK molecule is to stimulate the phosphorylation of Toll-like receptor 4 (TLR4) [64], which recognises bacterial lipopolysaccharide and induces inflammatory and immune responses [65]. This gene has been described as highly variable in cattle [66], and many *TLR4* polymorphisms and haplotypes have been associated with milk somatic cell count and susceptibility to mastitis [67]. Moreover, the SYK protein induces the recognition of pathogens and cell adhesion and platelet activation [65], and it also affects the proliferation of mammary epithelial cells at several stages of the milking cycle [66]. In the same enriched gene ontologies, we have detected the integrin subunit αM gene (*ITGAM* also known as *CD11b*) which maps to CHI25: 27,221,164–27,264,350 bp and encodes a receptor for lipopolysaccharide [68]. Signalling mediated by TLR4 activates the synthesis of ITGAM/CD11b, which is essential for the migration and adhesion of polymorphonuclear leukocytes to infection sites [68].

As previously indicated, genes related to chemotaxis were significantly enriched at the nominal level (*P*-value < 0.05) (Additional file 11: Table S9). This functional category is mainly represented by chemokine genes, such as chemokine ligand 19 (*CCL19*), 21 (*CCL21*), 24 (*CCL24*), 26 (*CCL26*) and 27 (*CCL27*). Chemokines are essential for the development of the innate immune response since they orchestrate and control the migration of the immune cells (macrophages, monocytes, neutrophils, etc.) to sites of infection [69]. During the first stages of mastitis, chemokines contribute to the stimulation of the cellular immune response against the invading pathogen until acute-phase proteins are expressed [70]. Marsland et al. [71] described how chemokines CCL19 and CCL21 participate in the maturation of dendritic cells, allowing them to leverage the T cell response. These chemokines also enhance the migration of leukocytes through lymph and blood circulation and stimulate the production of pathogen-induced proinflammatory cytokines [71]. In the gene set associated with inflammatory response (*P*-value = 0.03), we detected the interleukin 27 (*IL27*) gene which encodes a molecule with both pro and anti-inflammatory effects, thus enhancing the immune response and, at the same time, preventing tissue damage caused by inflammation [72]. Infections caused by Gram-negative bacteria induce IL27 production, and this cytokine interacts with monocytes increasing TLR4 expression and enhancing the LPS-induced inflammatory response [73]. Moreover, IL27 has an autocrine effect on macrophages and monocytes resulting in the amplification of the inflammatory response via cytokine secretion [72].

The genomic region displaying inbreeding depression for milk yield (MY210, MY240 and MY305) was significantly enriched at the nominal level (*P*-value < 0.05) with genes associated with multiple unrelated biological processes (Additional file 15: Table S13). The PPAR signalling pathway (*P*-value = 0.032) influences milk production and composition in cattle. Bai and collaborators [74] described an overrepresentation of genes from the PPAR signalling pathway in cows with high daily milk yield in comparison with low yielders. Besides, genes from this pathway are upregulated in cows in the lactation peak when compared to those in the dry period [75], suggesting a role of these genes not only in the determinism of fat composition [76] but also of milk yield.

## Conclusions

Murciano-Granadina goats display low levels of inbreeding (mean *F*_*ROH*_ = 0.053 ± 0.046), a finding consistent with the large census size and demographic history of this breed. Four genomic regions on CHI8:37,557,623–56,336,435, CHI8:75,115,244–93,894,055 bp, CHI25:82,419–7,143,084 and CHI25:21,429,255–28,572,340 bp were associated with inbreeding depression for lnSCC. Moreover, one region on CHI2:22,751,824–68,255,473 was consistently associated with inbreeding depression for three milk yield traits (MY210, MY240 and MY310). Genes encoding integrins, chemokines and pathogen recognition receptors, which play relevant roles in the elicitation of innate immune responses against microbes, mapped to regions associated with inbreeding depression for lnSCC. These results suggest that keeping inbreeding to a minimum, through an adequate reproductive management, might be a useful approach to decrease the incidence of mastitis in Murciano-Granadina goats.

## Supplementary Information

**Additional file 1: Table S1.** Number and density of SNPs per chromosome in a population of 1040 Murciano-Granadina goats genotyped with the Goat SNP50 BeadChip.

**Additional file 2: Figure S1.** Principal component analysis of 1040 Murciano-Granadina goats distributed in 15 farms (each farm is indicated with a different colour).

**Additional file 3: Figure S2.** Number of ROH per chromosome (represented as yellow bars, left axis) and the percentage of each chromosome covered by ROH (represented by a red line, right axis) in 1040 Murciano-Granadina goats.

**Additional file 4: Table S2.** Genomic regions associated with the top 1% of homozygosity for each SNP marker in a population of 1040 Murciano-Granadina goats genotyped with the Goat SNP50 BeadChip.

**Additional file 5: Table S3.** ROH hotspots identified in the genomes of 1040 Murciano-Granadina goats.

**Additional file 6: Table S4.** Genes mapping to regions consistently identified as ROH hotspots and regions with high homozygosity.

**Additional file 7: Table S5.** Functional enrichment analysis of genes mapping to regions consistently identified as ROH hotspots and regions with high homozygosity.

**Additional file 8: Table S6.** Inbreeding depression estimates (s.e: standard error) per goat chromosome (CHI) for the natural logarithm of the somatic cell count divided by 1000 (lnSCC) and their 95% confidence intervals (C.I).

**Additional file 9: Table S7.** Inbreeding depression estimates (s.e: standard error) for the natural logarithm of the somatic cell count divided by 1000 (lnSCC) in specific regions of goat chromosomes (CHI) 8 and 25 and their 95% confidence intervals (C.I).

**Additional file 10: Table S8.** Genes mapping to goat chromosome (CHI) 8 (37–56 Mb and 75–93 Mb) and 25 (0.082–7 Mb and 21–28 Mb) regions associated with inbreeding depression for the natural logarithm of the somatic cell count divided by 1000 (lnSCC).

**Additional file 11: Table S9.** Functional enrichment analysis of genes from the goat chromosome 8 (37–56 Mb and 75–93 Mb) and 25 (0.082–7 Mb and 21–28 Mb) regions associated with inbreeding depression for the natural logarithm of the somatic cell count divided by 1000 (lnSCC).

**Additional file 12: Table S10.** Inbreeding depression estimates (s.e: standard error) per goat chromosome (CHI) for milk yield at 210, 240 and 305 days (MY210, MY240 and MY305, measured in kg) and their 95% confidence intervals (C.I).

**Additional file 13: Table S11.** Inbreeding depression estimates (s.e: standard error) for milk yield at 210, 240 and 305 days (MY210, MY240 and MY305, measured in kg) in specific regions of goat chromosomes (CHI) 2 and their 95% confidence intervals (C.I).

**Additional file 14: Table S12.** Genes mapping to goat chromosome (CHI) 2: 22–68 Mb region associated with inbreeding depression for milk yield at 210, 240 and 305 days (MY210, MY240 and MY305).

**Additional file 15: Table S13.** Functional enrichment analysis of genes from the goat chromosome (CHI) 2: 22–68 Mb region associated with inbreeding depression for milk yield at 210, 240 and 305 days (MY210, MY240 and MY305).

## Data Availability

Goat SNP50 BeadChip genotypes and milk production phenotypes are accessible at Figshare (10.6084/m9.figshare.18095825).

## Abbreviations

IBD: Identical-by-descent
SNPs: Single nucleotide polymorphisms
IBS: Identity-by-state
ROH: Runs of homozygosity
CHI: Chromosomes
FP: Fat percentage
PP: Protein percentage
LP: Lactose percentage
PCA: Principal component analysis
DAVID: Annotation Visualization and Integration Discovery
GO: Gene ontology
REML: Restricted maximum likelihood
SYK: Spleen tyrosine kinase
