# Supplementary material for: Genomic patterns of homozygosity and inbreeding depression in Murciano-Granadina goats
Source: J Anim Sci Biotechnol. 2022 Mar 10;13:35. doi: 10.1186/s40104-022-00684-5 (PMC8908635; doi:10.1186/s40104-022-00684-5)
Supplement: Supplementary file 2 — Additional file 2: Figure S1. Principal component analysis of 1040 Murciano-Granadina goats distributed in 15 farms (each farm is indicated with a different colour). [file 40104_2022_684_MOESM2_ESM.docx]

**Figure S1.** Principal component analysis of 1,040 Murciano-Granadina goats distributed in 15 farms (each farm is indicated with a different colour).

**
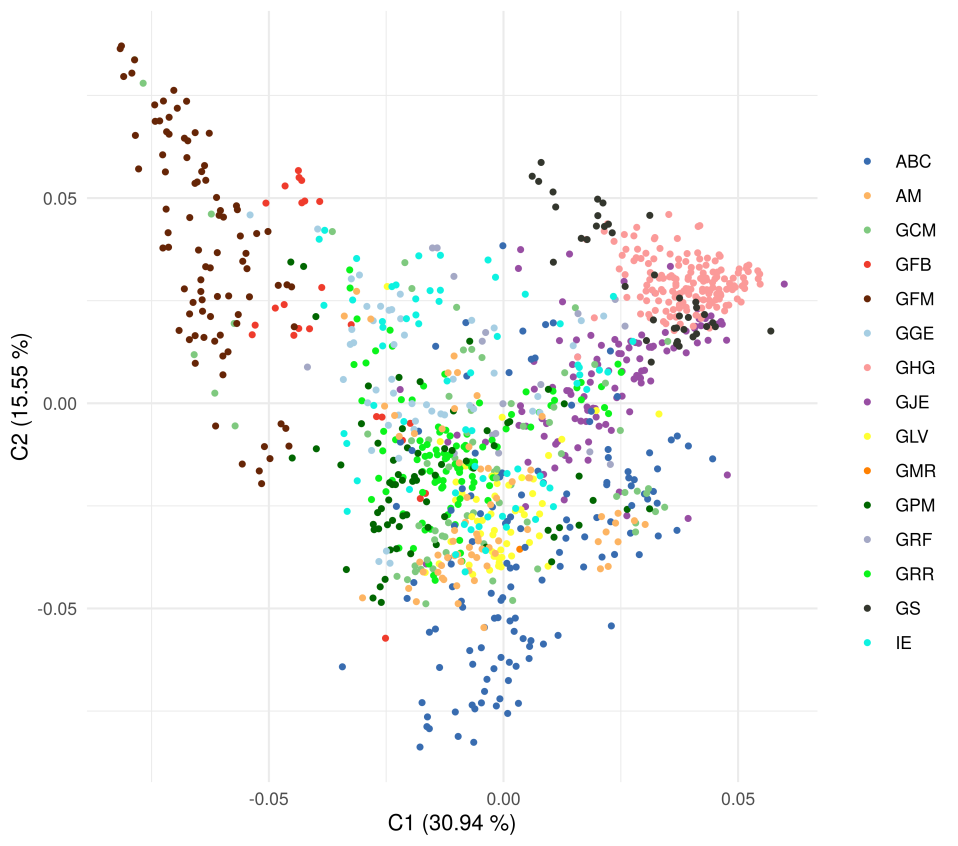
**
