# Supplementary material for: Genomic patterns of homozygosity and inbreeding depression in Murciano-Granadina goats
Source: J Anim Sci Biotechnol. 2022 Mar 10;13:35. doi: 10.1186/s40104-022-00684-5 (PMC8908635; doi:10.1186/s40104-022-00684-5)
Supplement: Supplementary file 3 — Additional file 3: Figure S2. Number of ROH per chromosome (represented as yellow bars, left axis) and the percentage of each chromosome covered by ROH (represented by a red line, right axis) in 1040 Murciano-Granadina goats. [file 40104_2022_684_MOESM3_ESM.docx]

**Figure S2.** Number of ROH per chromosome (represented as yellow bars, left axis) and the percentage of each chromosome covered by ROH (represented by a red line, right axis) in 1,040 Murciano-Granadina goats.

**
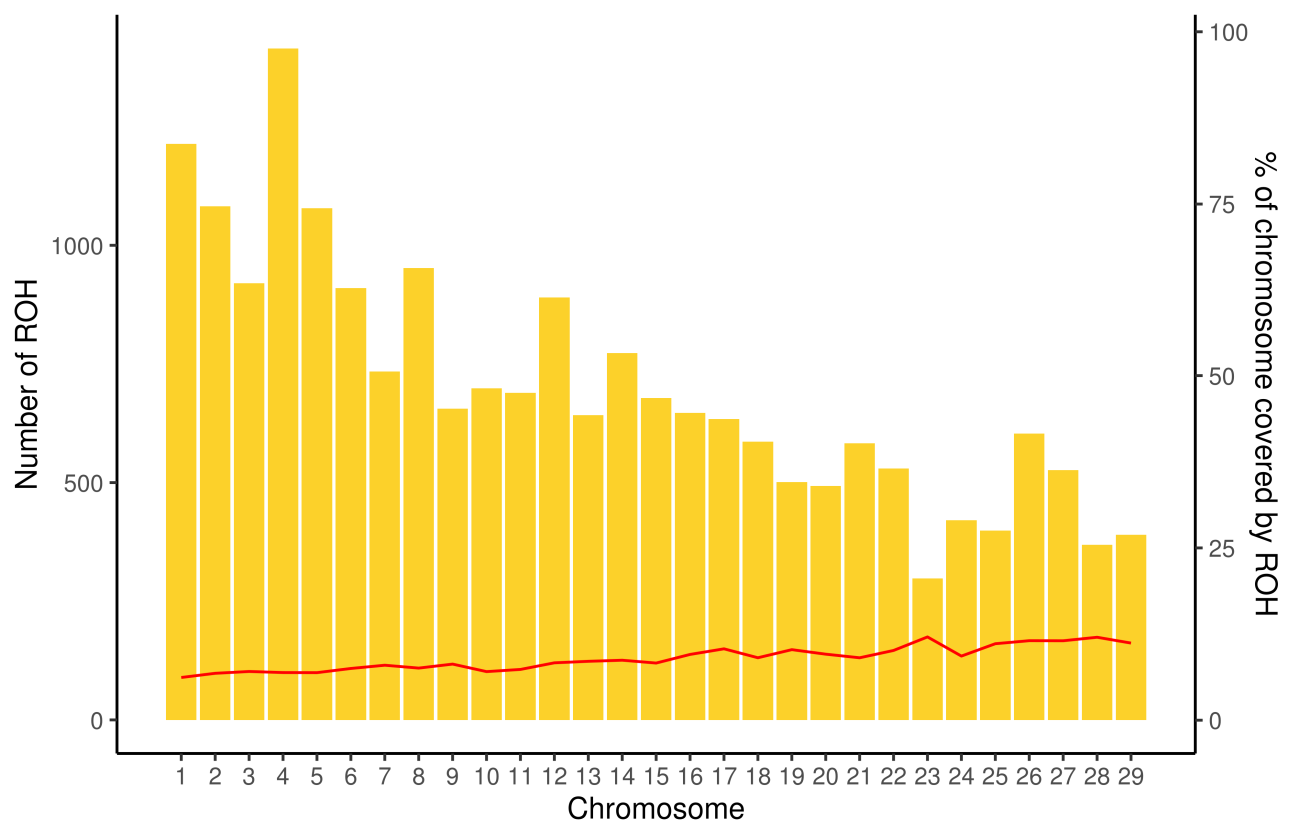
**
